# Guy's Hospital

**Published:** 1905-06-24

**Authors:** 


### GUY'S HOSPITAL.

The treasurer (Mr. H. Cosmo Bonsor), in a letter dated June 17th, recapitulates his appeal of March last. He proceeds:—

"Briefly, we asked for (1) £100,000, the balance of the funds sought to be raised in 1901 and urgently needed to carry Guy's through present straits. (2) Annual contributions of sufficient amount to enable the hospital to maintain its present work in continued efficiency.

"This appeal has been met in many quarters by most generous help. Towards the £100,000 required on capital account the response up to date is roundly £49,000. But in analysing this sum we find some striking and curious facts. One hundred and thirty-eight contributors, in sums ranging from £25 to £5,000, gave about £46,000. Two hundred and twenty contributors of sums under £25 subscribed rather more than £1,000, and the balance is provided by contributions from our Million Shilling Fund.

"The Press, ready as ever in the cause of suffering, has lent its aid to make this appeal generally known; it has been sent into the offices and homes of over 100,000 of the well-to-do classes in the metropolis, and in addition we have addressed personal letters to some thousands of those whom we considered likely to subscribe, yet we have received a direct response from only 358 persons. It is true that there have been a considerable number of contributions to the Million Shilling Fund, but this is almost entirely due to the personal efforts of our own doctors, students, and nurses.

"It is a fact that, with a few notable exceptions, Guy's Hospital has been saved to the public by the generosity and individual endeavours of a small group of old friends. For the balance of the necessary funds we must look to new friends, and in the firm belief that the English people will never allow a genuine need of the sick poor to remain unsatisfied, I again ask for space to make to them a simple and direct petition to exercise their privilege, and to aid this great charity, which is serving the nation so well.

"I would earnestly ask those who 'have this world's goods' to help us now, and also to remember Guy's Hospital in their wills. Experience has taught us how much the permanence and prosperity of our charitable foundations depend on this kindly forethought for the needs of others on the part of those who are no longer among us."

